# Molecular phylogeny and evolution of alcohol dehydrogenase (*Adh*) genes in legumes

**DOI:** 10.1186/1471-2229-5-6

**Published:** 2005-04-18

**Authors:** Tatsuya Fukuda, Jun Yokoyama, Toru Nakamura, In-Ja Song, Takuro Ito, Toshinori Ochiai, Akira Kanno, Toshiaki Kameya, Masayuki Maki

**Affiliations:** 1 Graduate School of Life Sciences, Tohoku University, Sendai 980-8577, Japan; 2 Graduate School of Life Sciences, Tohoku University, Sendai 980-8578, Japan; 3 Graduate School of Science, Hiroshima University, Hiroshima 739-8526, Japan

## Abstract

**Background:**

Nuclear genes determine the vast range of phenotypes that are responsible for the adaptive abilities of organisms in nature. Nevertheless, the evolutionary processes that generate the structures and functions of nuclear genes are only now be coming understood. The aim of our study is to isolate the alcohol dehydrogenase (*Adh*) genes in two distantly related legumes, and use these sequences to examine the molecular evolutionary history of this nuclear gene.

**Results:**

We isolated the expressed *Adh *genes from two species of legumes, *Sophora flavescens *Ait. and *Wisteria floribunda *DC., by a RT-PCR based approach and found a new *Adh *locus in addition to homologues of the *Adh *genes found previously in legumes. To examine the evolution of these genes, we compared the species and gene trees and found gene duplication of the *Adh *loci in the legumes occurred as an ancient event.

**Conclusion:**

This is the first report revealing that some legume species have at least two *Adh *gene loci belonging to separate clades. Phylogenetic analyses suggest that these genes resulted from relatively ancient duplication events.

## Background

The alcohol dehydrogenase (*Adh*) genes encode a glycolytic enzyme and have been characterized at the molecular level in a wide range of flowering plants [1-3] as well as in *Pinus banksiana*, a conifer species [4]. The ADH enzyme is essential for anaerobic metabolism [5-7]. In both *Arabidopsis thaliana *and maize, oxygen stress and cold stress induces transcription from the *Adh *promoters; in addition, dehydration induces *Adh *transcription in *A. thaliana *[5-7]. Flowering plant species generally possess two or three isozymes [8], although *A. thaliana *has a single *Adh *locus [9].

The *Adh *genes in *Arabidopsis thaliana *[10], *Arabidopsis gemmifera *[3] and *Leavenwortia *[11] in Brassicaceae, cottons [2], and grasses [12-15] have been subjected to molecular evolutionary studies. However, the broader evolutionary histories of the *Adh *genes in the angiosperms remain unclear since few studies have investigated the evolution of the *Adh *genes in a wide range of angiosperms. Recently, Small and Wendel [2] suggested that some *Adh *gene duplications may have predated the origin of each of the flowering plant families. However, the details of the gene duplications and deletions experienced by the *Adh *genes of most groups of the angiosperms remain unclear. Additional studies are needed to understand the evolutionary history of the *Adh *genes in various plant groups.

In the legume family (Fabaceae), the *Adh *genes have only been investigated in crop species such as *Glycine max *and *Pisum sativum*. The purpose of these studies was to determine the ADH structures and functions rather than to explore the evolutionary processes of the *Adh *genes [e.g., [16,17]], although these studies suggested that these legume species contained only a single *Adh *gene locus [16,17]. Previous phylogenetic analyses of the *Adh *genes from various flowering plants have revealed that all of the *Adh *genes in legume plants characterised to date constitute a monophyletic group [1,2]. In contrast, the *Adh *genes in Rosaceae, a family that is closely related to the Fabaceae [18,19], appear in two separate lineages of the gene tree, suggesting that a gene duplication event had occurred before the Rosaceae evolved [2]. Although these observations hint that the legume family may actually bear other *Adh *gene copies, this has not yet been investigated. Consequently, it remains unclear whether *Adh *gene duplication occurred during the evolution of the legume family.

Here, we report the isolation of *Adh *genes from two quite disparate legume species. We found that both of these species contain another *Adh *gene locus in addition to the locus that was identified in legume species previously. We also investigated the molecular evolutionary history of the *Adh *genes in this family to gain further understanding of the evolutionary dynamics of nuclear gene families.

## Results

### Isolation of the *Adh *genes in legume plants

**Figure 1:**
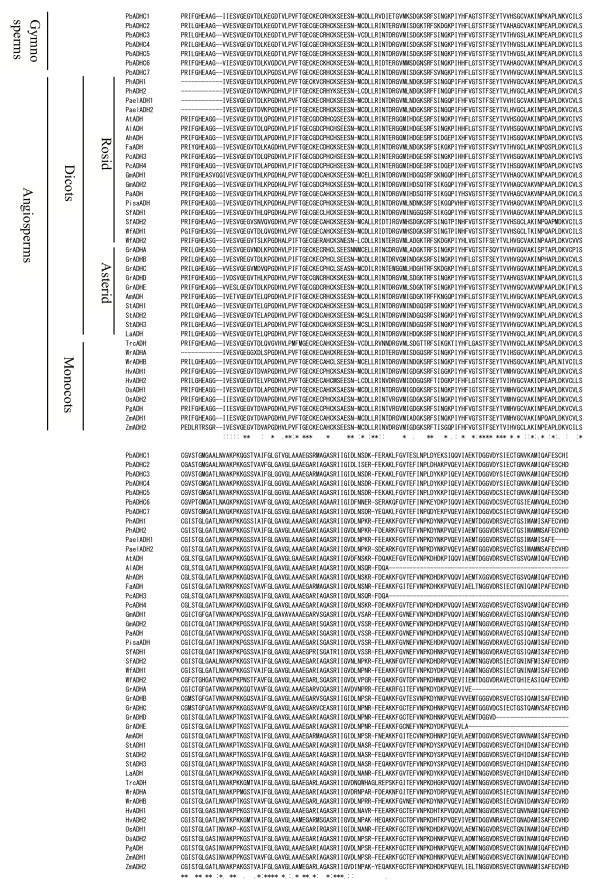
**Alignment of the predicted amino acid sequences from selected *Adh *gene representatives. **The lines indicate the groups that correspond to those in Figure 2.

Two *Adh *sequences were isolated from each of the two legume species examined in this study. The *Adh *genes isolated from *Sophora flavescens *Ait. were denoted *SfADH1 *and *SfADH2 *while the isolates from *Wisteria floribunda *DC. were denoted as *WfADH1 *and *WfADH2*. For these genes, 708 bp were sequenced. As shown in Fig. 1, this resulted in a predicted amino acid sequence consisting of 236 residues. The sequences determined in this study have been submitted to the DDBJ / EMBL / GenBank nucleotide sequence databases (Table 1). At the amino acid level, the homology among the *Adh *genes in the legume plants ranged from 70.7% to 91.8%.

### Phylogenetic analyses

**Figure 2:**
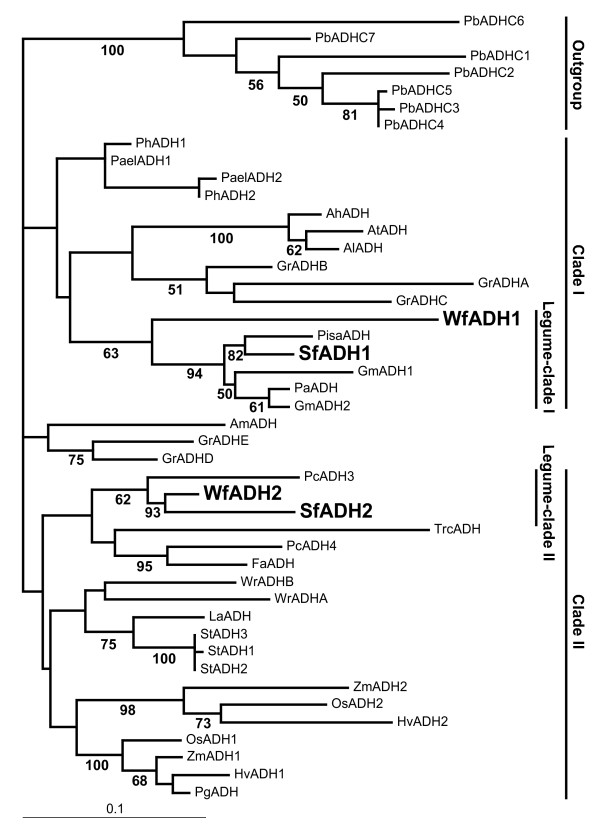
**The phylogenetic tree based on *Adh *gene sequences obtained by the maximum-likelihood method. **The log-likelihood of the best ML tree is -3981.05. The numbers below the branches are the bootstrap values of 50% or more support. The *Adh *genes from legumes roughly fall into two monophyletic clades that we denoted as Clade I and Clade II.

We conducted phylogenetic analyses of the *Adh *genes using seven sequences from *Pinus banksiana *(Pinaceae) as outgroups [4]. To determine the phylogenetic position of the legume *Adh *genes isolated in this study, we subjected their sequences to ML analysis by employing a data set including the previously published *Adh *gene family sequences from various phylogenetic groups [e.g., [1,2]]. Our resulting *Adh *gene tree roughly consisted of two monophyletic groups that we denoted "Clade I" and "Clade II" (Fig. 2). Clade I contains only *Adh *genes from dicots, while Clade II contains *Adh *genes from both dicots and monocots. The legume *Adh *genes isolated in this study appeared in two separate clusters, one in Clade I and the other in Clade II (Fig. 2). For convenience, we call these clusters " Legume-clade I" and " Legume-clade II". Legume-clade I contained the *SfADH1 *and *WfADH1 *sequences as well as previously published *Adh *genes sequences from the legumes *Glycine max*, *Pisum sativum*, *Phaseolus actifolius *and *Trifolium repens *(Fig. 2). Legume-clade II consisted of only the *SfADH2 *and *WfADH2 *sequences and was located far from the other legume *Adh *sequences (Fig. 2). None of the other legume *Adh *sequences that have been published previously fell into Legume-clade II. However, the *Adh *gene in *Pyrus communis *(Rosaceae), which belongs to the family that is closely related to the Fabaceae [e.g. [18,19]], occurred at the sister position to Legume-clade II.

**Figure 3:**
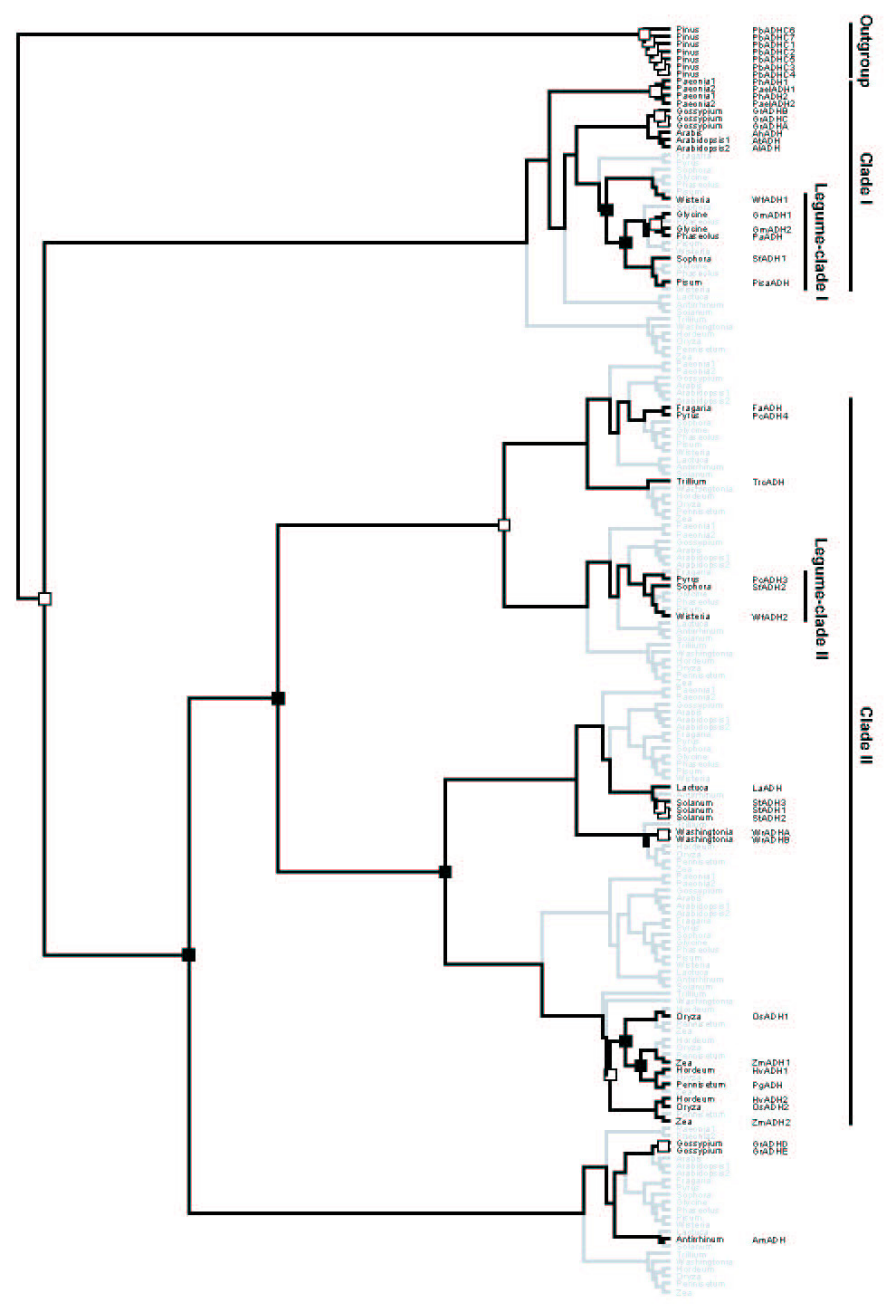
**Part of the simplest reconciled tree that has the lowest number of duplication and deletion events. **The reconciled tree involves 24 gene duplications and 44 gene losses for a total cost of 68, and requires 40 deep coalescenses. The solid boxes indicate gene duplications that were inferred on the basis of mismatches between the gene tree and the species tree. The open boxes indicate those duplications that required multiple copies of *Adh *genes within the same species. The gray lines indicate the lineages that are presumed to be lost after the duplications or were not found in our analysis.

GeneTree analysis using the *Adh *gene sequences suggested that the legume *Adh *genes were duplicated before and after the angiosperms diversified (Fig. 3). This indicates that the *Adh *genes in Clade II have undergone more duplication events than those in Clade I (Fig. 3).

## Discussion

### Molecular phylogeny of the *Adh *sequences in legume plants

Although a previous study detected a monophyletic group of *Adh *genes in legumes [1], we found additional legume *Adh *genes that were related more distantly to the previously detected legume *Adh *genes. This is the first report showing that there are two *Adh *lineages in legume plants, each of which belongs to quite separate clades denoted as Legume-clade I and II, which themselves fall into distinct clades denoted as Clade I and II (Fig. 2). Notably, the *Adh *genes belonging to Legume-clade I are closely related to the *Arabidopsis thaliana *gene in Clade I (Fig. 2). *Arabidopsis thaliana *has a single *Adh *locus and transcription from its promoter increases under cold and oxygen stress [5-7]. Thus, the legume *Adh *genes in Legume-clade I may have similar functions to that of the *A. thaliana *gene. Our study also revealed that the legume *Adh *genes belonging to Legume-clade II form a sister group to the *Adh *gene isolated from *Pyrus communis *in Rosaceae (Fig. 2), which is a closely related family to the Fabaceae in the angiosperm phylogeny [20,21].

The function of the *Adh *gene in maize is also similar to that of *A. thaliana *[5-7]. Thus, our phylogenetic result suggests that function is the plesiomorphic character of the *Adh *gene family (Fig. 2). On the other hand, Clade II consists of many genes of both monocots and dicots, suggesting that the functions of the *Adh *genes in this clade may be more diversified due to the accumulation of many mutations during the course of angiosperm diversification that alter the primary structure of the ADH proteins. However, our phylogenetic analyses failed to indicate whether the genes in the Legume-clade II are orthologues or paralogues of the *Adh *gene in maize (Fig. 2). Thus, the function of the *Adh *genes in Legume-clade II remains unclear.

### Gene duplication of *Adh *genes in legume plants

This study revealed the complicated evolution of the *Adh *gene family that occurred during the course of plant diversification. In our study, the phylogenic tree resulting from GeneTree analysis showed that some *Adh *genes in flowering plants evolved in complex manner that included several duplication events (Fig. 3). Duplication events in *Adh *genes have also been detected in other plant groups at various evolutionary levels. For example, Sang et al. [22] showed that diploid species of *Paeonia *(Paeoniaceae) had two or three *Adh *sequences and that repeated duplication or deletion events occurred after the diversification of this genus. Small and Wendel [2] analyzed *Adh *genes in *Gossypium *(Malvaceae) in great detail and found that these *Adh *sequences (denoted as *GrADHA*, *GrADHB*, *GrADHC*, *GrADHD*, and *GrADHE*) had experienced duplication events both before and after the divergence in *Gossypium*. Consistent with this, our GeneTree analysis revealed that in legumes, duplication of *Adh *genes occurred before the legume diverged, since the two quite distinct legumes *Wisteria floribunda *and *Sophora flavescens *have paralogous genes in each of two clades (Fig. 3), although all previously known *Adh *genes in legume plants such as *Glycine max*, *Pisum sativum *and *Phaseolus actifolius *belong only to Legume-clade I.

Why were additional *Adh *loci not found in other legumes? It is possible that the expression of the Legume-clade II *Adh *genes in *Glycine max*, *Pisum sativum *and *Phaseolus actifolius Adh *genes is limited to a specific developmental period or organ. Further analysis of *Adh *mRNA expression during various developmental phases and in different organs of these plants, such as roots, stems and fruits, may reveal the presence of an additional *Adh *gene in these species. Another possibility is that orthologues of the Legume-clade II *Adh *gene in the previously examined species have lost their function. Additional investigations throughout the legume family are needed to test this hypothesis.

## Conclusion

Duplicated genes arise frequently in eukaryotic genomes through local events that generate tandem duplications, large-scale events that duplicate chromosomal regions or entire chromosomes, or genome-wide events that result in complete genome duplication [23]. Indeed, the existence of multigene families is evidence of the repeated gene duplication that has occurred over the history of life. One of the examples of the comprehensive analysis of gene duplication events in plants is the study of the MADS-box gene family. This gene family, which plays a central role in the morphogenesis of plant reproductive organs such as ovules and flowers, had experienced duplication events before the origin of angiosperms [24]. Moreover, some specific functions were gained through duplication events that took place after the diversification of flowering plants [24]. Thus, gene duplication has long been recognized as an important mechanism for the creation of new gene functions [25-27]. It is likely that each of the *Adh *genes in the legumes that were identified in the present study would have been subjected to different selective pressures over a long period. To determine whether this resulted in new functions, functional analyses of the legume *Adh *genes in each clade will have to be performed in the future.

## Methods

### Plant materials

In this study, we chose *Sophora flavescens *Ait. and *Wisteria floribunda *DC. from the legume family (Fabaceae). They belong to different subfamilies or tribes in the traditional classification [28]. They also fall into different phylogenetic groups in the phylogenetic tree constructed using legume *rbcL *sequences [29,30]. We also used tissues from *Antirrhinum majus *L. (Scrophulariaceae) and *Trillium camtschatcense *Ker-Gawl. (Trilliaceae). Flowers and leaf tissues were collected from the experimental garden of Tohoku University and native individuals of these species in the field. Vouchers for all species used in this study are listed in Table 2 and have been deposited in the Herbarium, Graduate School of Science, Tohoku University (TUS).

### Isolation of RNA

Total mRNA was isolated according to the modified protocol of Hong et al. [31]. Thus, 3 g of flowers and leaf tissues were homogenized for 2 min with 3 volumes of detergent buffer containing 10 mM Tris-HCl (pH8.8), 50 mM NaCl, 6% (w/v) p-aminosalicylic acid, 2% (w/v) triisopropylnaphtalensulfonic acid, and 6% (v/v) 1-butanol. The homogenates were extracted three times with an equal volume of phenol/chloroform/isoamyl alcohol (25:24:1, v/v/v) with vigorous shaking. The final aqueous phase was collected and the total RNA was precipitated with ethanol and 3 M sodium acetate on ice for 1 hr. The total RNA was then treated with Oligotex-dT30 (TAKARA, Japan) to purify the poly(A) RNA.

### Cloning and sequence analysis

Single-stranded cDNA was synthesized by priming with the random 9-mer or the oligo-dT adaptor primer (TAKARA). The cDNA was amplified by PCR in a 50 μL reaction volume containing approximately 50-ng total DNA, 10-mmol/L Tris-HCl buffer (pH 8.3) with 50-mmol/L KCl and 1.5-mmol/L MgCl_2_, 0.2-mmol/L of each dNTP, 1.25 units *Taq *DNA polymerase (TAKARA) and 0.5-μmol/L of each primer. The primers used have been published previously and are denoted as ADH-F1, ADH-R1 and ADH-R2 [22]. A degenerate primer was also used (LADH-1F1: 5'-ATATTTGGTCAYGAAGCTGG-3'). This primer was designed on the basis of the conserved region of *Adh*, which was determined by comparing the published sequences of *Adh *[22]. We carried out PCR with the following thermocycle protocol: (94°C, 2 min) × 1 cycle; (94°C; 30 sec, 50°C; 30 sec, 72°C; 120 sec) × 45 cycles; (72°C; 15 min) × 1 cycle. After the amplification, the reaction mixtures were subjected to electrophoresis in 1.5% low-melting-temperature agarose gels and the amplified products were purified. The purified PCR products were then cloned using the TA cloning kit (Invitrogen). Plasmids containing the cloned fragments were isolated by the alkali method and digested with *Eco*RI. Plasmids containing fragments less than 1.5 kb in size were selected and sequenced using the Thermo Sequence II dye terminator cycle sequencing premix kit (Amersham Pharmasia Biotech) or the BigDye Terminator cycle sequencing premix kit (Applied Biosystems) with the Model 373A or 310 automated sequencer (Applied Biosystems) according to the manufacturer's instructions.

### Phylogenetic analysis

The sequences of the *Adh *genes used in this study were obtained from the GenBank/EMBL/DDBJ database (Table 1). The predicted amino acid sequences were aligned using CLUSTAL X [32] based on the GONNET protein weight matrix. The phylogenetic relationships between the genes were analyzed using the maximum-likelihood (ML) method. For the ML analyses, we used the PROTML program of PHYLIP version 3.6 [33]. We employed the JTT model of amino acid substitution. All indels were counted as missing. We performed ten random sequence addition searches using the J option and global branch swapping using the G option to isolate the ML tree with the best log-likelihood. In addition, we performed bootstrap analysis with 100 replications.

To infer the evolutionary events affecting the *Adh *genes, an analysis using GeneTree ver. 1.3 [34] was conducted, as described by Fukuda et al. [35]. The fully-resolved species tree used in the analysis was constructed on the basis of the previously published *rbcL *sequences in chloroplast DNA; the tree is considered to indicate the evolutionary relationships of the plants from which the *Adh *genes studied in this study were isolated [28]. The ML tree with the highest log-likelihood was used for the gene tree. Both gene duplications and losses were considered to reconcile the gene tree with the species tree. Gene lineages that do not coalesce on each branch of the species tree were counted as deep coalescence [36].

## Authors' contributions

TF carried out the molecular genetic studies, IS and TN participated in the sequence alignment, and IT and TO drafted the manuscript. JY participated in the design of the study and performed the phylogenetic analysis. AK, TK and MM conceived of the study, participated in its design and coordination, and helped to draft the manuscript. All authors read and approved the final manuscript.

## Figures and Tables

**Table 1:** List of accession numbers used in this study. List of taxa and source of this study.

Phylogenetic group			
Family			
Species	ADH locus	Acc. No.	References
Gymnosperms			
Pinaceae			
*Pinus banksiana*	*PbADHC1*	U48366	Perry and Furnier 1996
	*PbADHC2*	U48373	Perry and Furnier 1996
	*PbADHC3*	U48368	Perry and Furnier 1996
	*PbADHC4*	U48369	Perry and Furnier 1996
	*PbADHC5*	U48370	Perry and Furnier 1996
	*PbADHC6*	U48371	Perry and Furnier 1996
	*PbADHC7*	U48372	Perry and Furnier 1996
Angiosperms			
Dicotyledon			
Rosid			
Paeoniaceae			
*Paeonia humulis*	*PhADH1*	AF126226	Sang and Zhang 1999
	*PhADH2*	AF126232	Sang and Zhang 1999
*Paeonia lutea*	*PaelADH1*	AF009042	Sang et al. 1997
	*PaelADH2*	AF009057	Sang et al. 1997
Brassicaceae			
*Arabidopsis thaliana*	*AtADH*	D63464	Miyashita et al. 1996
*Arabidopsis lyrata*	*AlADH*	AJ251284	Savolainen et al. 2000
*Arabis hirsuta*	*AhADH*	AB015502	Miyashita et al. 1998
Rosaceae			
*Fragaria x ananassa*	*FaADH*	X15588	Wolyn and Jelenkovic 1990
*Pyrus communis*	*PcADH3*	AF031899	Chervin et al. 1999
	*PcADH4*	AF031900	Chervin et al. 1999
Fabaceae			
*Glycine max*	*GmADH1*	AF079058	Preiszner et al. unpubl.
	*GmADH2*	AF079499	Preiszner et al. unpubl
*Phaseolus acutifolius*	*PaADH*	Z23171	Garvin et al. 1994
*Pisum sativum*	*PisaADH*	X06281	Llewellyn et al. 1987
*Sophora flavescens*	*SfADH1*	AB191335	This study
	*SfADH2*	AB191336	This study
*Wisteria floribunda*	*WfADH1*	AB191337	This study
	*WfADH2*	AB191338	This study
Malvaceae			
*Gossypium raimondii*	*GrADHA*	AF182116	Small and Wendel 2000
	*GrADHB*	AF226635	Small and Wendel 2000
	*GrADHC*	AF036568	Small and Wendel 2000
	*GrADHD*	AF250203	Small and Wendel 2000
	*GrADHE*	AF250208	Small and Wendel 2000
Asterid			
Scrophulariaceae			
*Antirrhinum majus*	*AmADH*	AB191334	This study
Solanaceae			
*Solanum tuberosum*	*StADH1*	M25154	Matton et al. unpubl.
	*StADH2*	M25153	Matton et al. unpubl.
	*StADH3*	M25152	Matton et al. unpubl.
Asteraceae			
*Lactuca sativa*	*LaADH*	D44449	Toyomasu et al. 1995
Monocotyledon			
Trilliaceae			
*Trillium camtschatcense*	*TrcADH*	AB191339	This study
Arecaceae			
*Washingtonia robusta*	*WrADHA*	U65973	Morton et al. 1996
	*WrADHB*	U65972	Morton et al. 1996
Poaceae			
*Hordeum vulgare*	*HvADH1*	X07774	Good et al. 1988
	*HvADH2*	X12733	Trick et al. 1988
*Oryza sativa*	*OsADH1*	X16296	Xie and Wu 1989
	*OsADH2*	X16297	Xie and Wu 1989
*Pennisetum glaucum*	*PgADH*	X16547	Bui et al. 1990
*Zea mays*	*ZmADH1*	M32984	Osterman and Dennis 1989
	*ZmADH2*	X02915	Dennis et al. 1985

**Table 2:** List of taxa from which *Adh *was isolated in this study and source of plant materials. List of taxa that *Adh *was isolated in this study and source of plant materials.

Taxa	Family	Locality	Collecters
*Sophora flavescens*	Fabaceae	Japan: Miyagi Pref., Sendai	Fukuda 99081
*Wisteria floribunda*	Fabaceae	Japan: Miyagi Pref., Shiroishi	Fukuda and Yoshida 99051
*Antirrhinum majus*	Scrophulariaceae	cultivated in Tohoku University	Fukuda 98031
*Trillium camtschatcense*	Trilliaceae	Japan: Hokkaido Pref., Hidaka, Shizunai	Fukuda and Yokoyama 0404301
